# Thoracic Radionecrosis Following Repeated Cardiac Catheterization

**DOI:** 10.1155/2011/201839

**Published:** 2010-12-15

**Authors:** Borut Banic, Bernhard Meier, Andrej Banic, Christian Weinand

**Affiliations:** ^1^Department of Plastic and Reconstructive Surgery, University Hospital Inselspital, University of Bern, CH-3010 Bern, Switzerland; ^2^Department of Cardiology, Bern University Hospital Inselspital, University of Bern, CH-3010 Bern, Switzerland

## Abstract

Radiodermatitis is a known complication in patients having undergone radiotherapy. It usually appears 2 to 5 years after irradiation. We are reporting on a case of radiodermatitis that occurred within months after coronary dilatation and stenting. It started with painful swelling, followed by a typical appearance on the skin surface. Histological finding confirmed the diagnosis. However, magnetic resonance imaging showed changes in the subcutaneous tissue extending into the ribs. A radical debridement was performed including removal of a partially necrotic 4th rib. The defect was closed with a latissimus dorsi transposition flap. Our findings are compared with the literature reports.

## 1. Introduction

In the past 20 years there has been an enormous growth in the number of diagnostic and interventional procedures using fluoroscopy. It is known that cardiac catheterization procedures expose the patient to significant levels of radiation [1, 2]. However, until now, protection guidelines against radiation hazards associated with cardiac catheterization remain mostly on staff exposure [3].

Cutaneous side effects of X-rays have been observed and reported on since 1899 [4]. However, until recently little attention was paid to dermal radiation injury, following cardiac catheterization [5] for which there are no legally defined upper limits in Switzerland. Typical are skin lesions such as ulcerations and transformation of these lesions into squamous cell carcinoma. Specifically, threshold doses for the development of erythema, permanent epilation, moist desquamation, and necrosis are reported to be 3–10, 7–10, 12–25, and 25 gray, respectively [6, 7]. When these threshold doses for dermal injuries are surpassed, further injury of the underlying tissue results. The sequelae of radiation injuries due to higher doses than 25 gray are not known so far. We report on a case of transthoracic necrosis of skin and bone of a patient, who underwent cardiac catheterization with prolonged ionization of significantly more than 25 gray.

## 2. Case Report

A 59-year-old patient presented with typical chest pain to the cardiology service in May 2002 and underwent cardiac stenting of a highly stenotic lesion in the right coronary artery (RCA). The catheterization procedure entailed a total of 22 minutes of fluoroscopy. Six months later the patient presented with recurrent chest pain. Repeat angiography showed an occlusion of the RCA. Recanalization was attempted but was not successful. Fluoroscopy time during this procedure was 47 minutes. Due to persistent angina pectoris angiography was repeated 2 days later when the RCA could be recanalized and 2 stents were implanted successfully. Fluoroscopy time was 78 minutes. The total estimated radiation dose was well over 25 gray. 

**Figure 1:**
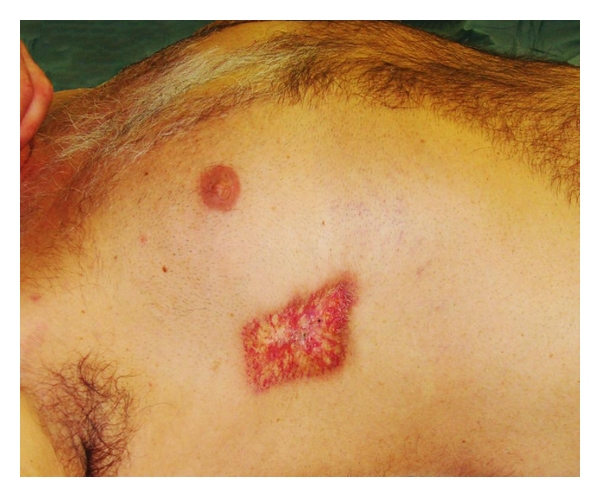
Radiodermatitis measuring 8 × 5 cm occurred within months after coronary dilatation and stenting.

Two months later the patient complained about a painful lesion on the right chest wall overlying the 4th rib. Physical exam revealed a 7 × 8 cm poikilodermal erythema. The lesion persisted in spite of treatment by dermatologists over 5 years (Figure 1).

**Figure 2:**
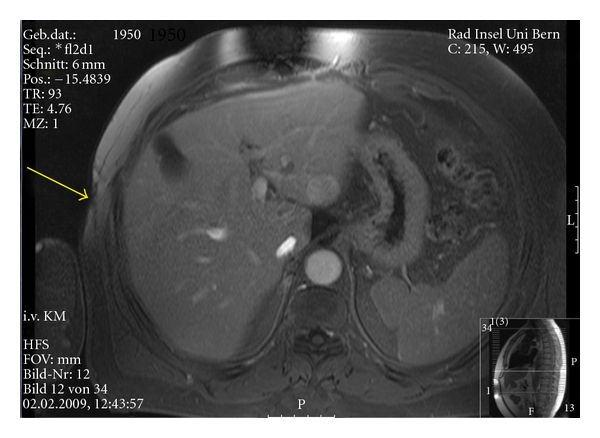
Magnetic resonance imaging (MRI) showing changes in the subcutaneous tissue extending to the ribs.

Magnetic resonance imaging (MRI) confirmed the suspected radionecrosis of the skin as well as necrosis of subcutaneous tissues (Figure 2). 

**Figure 3:**
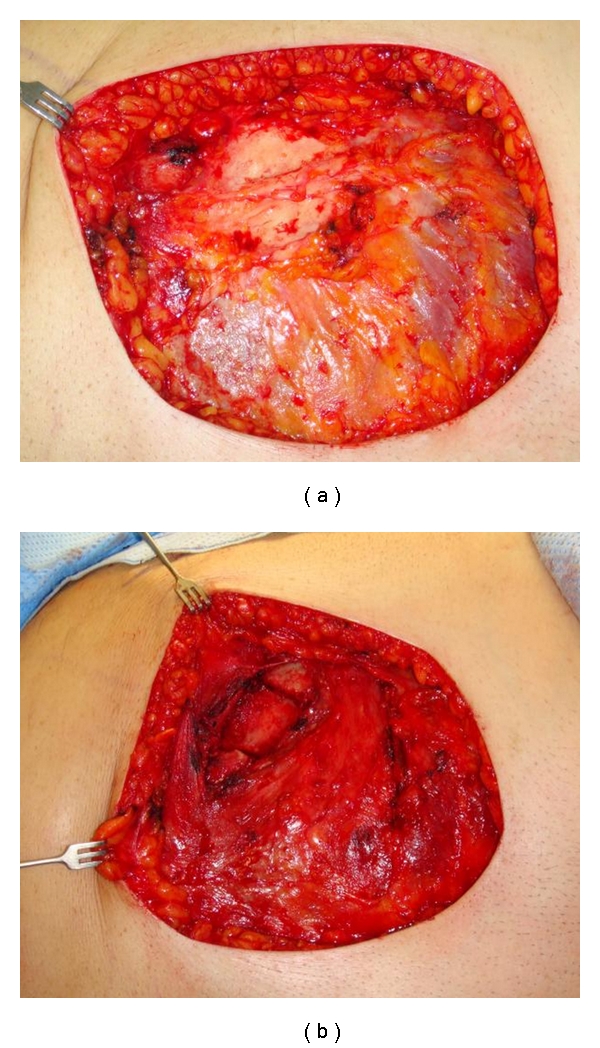
(a) Necrotic muscle covering the rib after the removal of radiodermatitis. (b) Situs after partial removal of the 4th rib.

In February 2009 the patient presented to the Department of Plastic and Reconstructive Surgery in our hospital. Indication was set for an excision of the lesion and defect coverage with a flap. During the operation, next to the soft tissues, the central portion of the 4th rib was found to be necrotic and had to be removed (Figures 3(a) and 3(b)). The excised tissue was sent for histopathological evaluation. The resulting defect, now measuring 15 × 10 cm, was subsequently covered by a pedicled myocutaneous latissimus dorsi flap from the right side.

**Figure 4:**
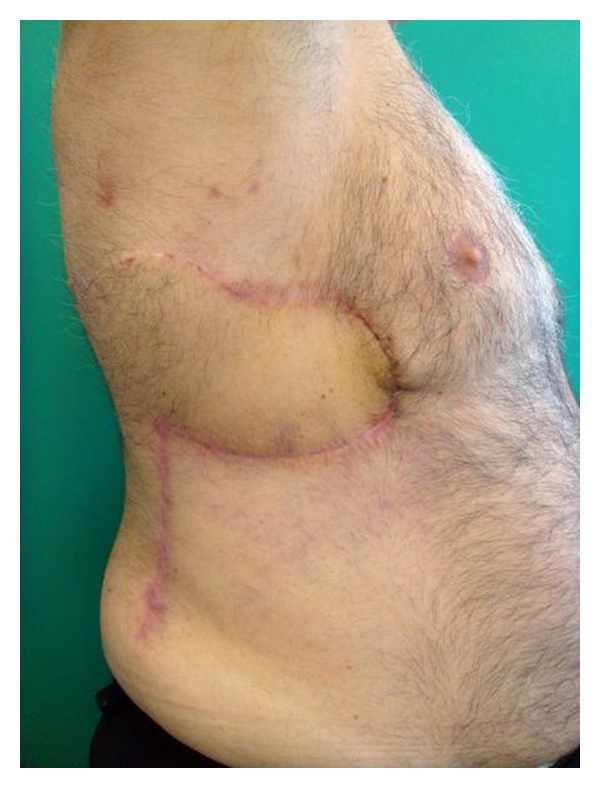
Wound healed after reconstruction of the defect with a latissimus dorsi transposition flap.

During the course of hospitalization the flap healed without complications (Figure 4). The histological results confirmed the radionecrosis.

Anticoagulation had to be stopped preoperatively. After the successful operation, the patient started to complain of chest pain. Repeat angiography showed a recurrent stenosis within the implanted stents. The stenosis was dilated again and 2 additional stents were implanted with a good result. Fluoroscopy time was 9 minutes. The defect coverage of the excised chest tissue healed well, and the patient did not develop any further skin changes.

## 3. Discussion

Although there has been an enormous growth of cardiac catheterization procedures in the last 20 years, there are no regulations that limit the radiation dose to the patient. Radiodermatitis as a complication of therapeutic use of ionizing radiation such as catheter-based coronary interventions is described only in a few case reports [8, 9]. Cutaneous side effects are dose dependent and can develop months or even years after radiation exposure. The cumulative dose necessary to induce chronic skin changes is estimated to be above 10 to 12 gray [3]. The cardiac catheterization ionizing radiation exposure rate can reach 0.008–0.016 coulomb/kg × minute, during a short and relatively constant time [1, 5]. A routine cardiac catheterization procedure exposes a patient to an average radiation dose of 2.5 gray whereas percutaneous interventions result in an average dose of 6.4 gray [4]. Our patient received a cumulative dose much higher than 25 Grey that led to skin changes already two months after the last exposure. 

To date, 9 cases of radiation dermatitis have been reported in the literature [4, 6, 8–12]. However, such extensive tissue necrosis has not been reported. In most cases of radiodermatitis, patients had undergone repeat cardiac catheterization procedures due to complex and anatomical challenging cases. However, most cases lack information about exposure time and resulting radiation. In our case the patient underwent several catheterization procedures in November and December 2002, the first lasting 21, the second 47, and the third 78 minutes. This totals an exposure time in 2002 of 157 minutes. Another 9 minutes were added in 2003. The procedures had been described as difficult because of the tortuosity of the RCA. Therefore, the most radiointense lateral projection (longest possible intrabody trajectory) had to be used most of the time. This also explained the localization of the skin lesion at the lateral entry side of the chest. 

The patient was transferred to our Department of Plastic and Reconstructive Surgery for excision of the lesion in 2008 after 5 years of continuous dermatological treatment. The tissue necrosis that was encountered intra-operatively was deeper than previously seen on the MRI. The resulting defect was closed using a latissimus dorsi myocutaneous transposition flap. The patient recovered well after plastic surgery and followup showed good healing of the soft tissue over the defect. 

In summary we present a case of a large and deep thoracic tissue necrosis after cardiac catheterization. Its early onset less than two months after the last catheterization indicates that the radiation dose the tissues received was well above the threshold of 25 Grey. Although the patient was successfully treated with a late excision and soft tissue coverage, we suggest that an early excision within several weeks after the onset of the radiodermatitis should be performed.
